# Effectiveness of Pricing Strategies on French Fries and Fruit Purchases among University Students: Results from an On-Campus Restaurant Experiment

**DOI:** 10.1371/journal.pone.0165298

**Published:** 2016-11-03

**Authors:** Tom Deliens, Benedicte Deforche, Lieven Annemans, Ilse De Bourdeaudhuij, Peter Clarys

**Affiliations:** 1 Department of Movement and Sport Sciences, Vrije Universiteit Brussel, Brussels, Belgium; 2 Department of Public Health, Ghent University, Ghent, Belgium; 3 Department of Movement and Sports Sciences, Ghent University, Ghent, Belgium; Indiana University Bloomington, UNITED STATES

## Abstract

**Objectives:**

This study examined the effect of a 10 and 20% meal price increase when choosing French fries and a 10 and 20% meal price reduction when choosing fruit for dessert on university students’ purchasing behaviour in an on-campus restaurant. The moderating effect of gender was also investigated. Secondly, this study aimed at gaining further insight into reasons why these price manipulations did or did not change students’ purchasing behaviour.

**Materials and Methods:**

This two-phased mixed-methods study was conducted in a Belgian on-campus university restaurant with approximately 1200 to 1300 student visitors per day. In a first phase (French fries experiment), data were collected during a control week (no price manipulation) and two separate intervention weeks (10 and 20% meal price increase when students chose French fries). In a second phase (fruit experiment), following the same protocol but carried out a few weeks later, meal prices were reduced by 10 and 20% when students chose fruit for dessert. French fries and fruit sale counts relative to the total number of items sold were used as outcome measure. Short interviews were conducted in convenient subsamples of student customers to assess influences on food choice.

**Key findings:**

Increasing the meal price by 10 and 20% when choosing French fries was associated with respective 10.9 and 21.8% absolute reductions in French fries purchases, while reducing the meal price by 10 and 20% when choosing fruit for dessert was associated with absolute increases in fruit purchases of respectively 25.1 and 42.4% (all *p*<0.001). No moderating effect of gender was detected. Besides price, food/taste preference, eating habits, health, availability and accessibility, and body satisfaction influenced students’ food choices, with taste being the most frequently mentioned factor.

**Significance:**

Pricing may be a promising strategy to improve university students’ eating behaviour. The likelihood of intervention success may increase when combining pricing strategies with offering healthy, tasty and meal matching starchy alternatives to French fries and offering a variety of fresh and appealing fruits.

## Introduction

The years at college or university is a period characterized by changes in eating behaviour [1–3]. For many students, dietary patterns do not meet the recommended guidelines which may result in weight gain [4, 5]. A significant positive association was found between the frequency of eating at the on-campus university restaurant of the Vrije Universiteit Brussel (Brussels, Belgium) and increases in body mass index (BMI) and fat%, suggesting that Belgian students eating at the on-campus restaurant might make more unhealthy food choices [4]. As unhealthy dietary intake may increase metabolic health risks (e.g. cardiovascular diseases, diabetes mellitus), it has been advocated that universities need to take an active role in designing and implementing food-related health promotion interventions [1, 6].

One strategy to help students make healthier food choices comprises price manipulations. A review by Andreyeva and colleagues [7] showed that price elasticity is highest for food away from home, soft drinks, juice, meats, and fruit, which means that, if the price of these foods increases, consumption will decrease, and vice versa. According to this economic theory, price adjustments could encourage people to make more healthy food choices. A number of recent reviews showed that both price taxations and subsidies can modify the purchase and consumption of targeted foods in various settings and populations [8–10]. To be successful, food taxes and subsidies should be a minimum of 10 to 15% [10]. Moreover, Epstein and colleagues [8] argued that, without the knowledge that a price manipulation is implemented, the price change may not influence behaviour. Hence, along with pricing strategies, people should be informed and be fully aware of the price adjustments in order to maximise their effect on purchasing [8].

Although food price is shown to be an important determinant of students’ eating behaviour [3, 11, 12], experimental research on the effects of price adjustments on students’ consumption or purchasing behaviour is scarce. Cardenas and colleagues [13] combined a fruit price reduction of 33% with repositioning of fruits and point-of-purchase messages in a Peruvian university cafeteria. Although the latter study (including students as well as non-students) suggested that male cafeteria consumers may be more susceptible to price adjustments than their female counterparts, no effect on fruit purchases was found among the student consumers [13]. In another study, using taxation of high-calorie foods, it was concluded that a price increase of ≥25% on high-calorie foods successfully made students buy fewer calories [14]. As taxation of ≥25% is quite drastic, the authors suggested to further investigate the effectiveness of smaller taxes. Besides the latter two studies, no interventions aiming at improving university students’ food choices by adjusting food prices were found. Moreover, previous intervention studies using price manipulations did not provide any information about why the price adjustments were or were not effective. Such information is crucial when designing interventions aiming at improving university students’ eating behaviour.

Therefore, the purpose of the present study was to examine the effect of a 10 and 20% meal price increase when choosing French fries (unhealthy food product) and a 10 and 20% meal price reduction when choosing fruit (healthy food product) on Belgian university students’ purchasing behaviour. The moderating effect of gender was also investigated. Secondly, using short interviews this study aimed to gain further insight into the reasons why such price manipulations did or did not change students’ purchasing behaviour.

## Materials and Methods

### Participants and setting

The present study was conducted in the on-campus restaurant of the Vrije Universiteit Brussel (Brussels, Belgium). The study sample consisted of all university students ordering a meal during lunch break. Approximately 1200 to 1300 university students visit the campus restaurant every day (Monday to Friday, from 11:30 AM to 1:45 PM). University staff members and other non-students (who also eat lunch at the on-campus restaurant) were excluded from the study. The university restaurant operates by a free flow system which allows students to choose between six different types of menus (‘menu 1’, ‘menu 2’, ‘health-menu’, ‘vegetarian-menu’, ‘pasta menu’, ‘wok menu’) or the salad bar. For the first four menus students are free to choose between French fries, (mashed) potatoes, or rice. For students, a typical menu includes soup of the day, meal and dessert (for which students may choose between fruit, yoghurt, pudding, cookies, or other kinds of dessert like ice cream) and costs € 5. An overview of starchy products and desserts (including nutritional values) offered at the on-campus restaurant is displayed in Table 1.

**Table 1:** Nutritional values (per portion) of starchy products and desserts offered at the on-campus restaurant.

Food product	Energy (kcal)	Carbohydrates (g)	Sugars (g)	Fat (g)	Saturated fat (g)	Salt (g)
*Starchy products*						
French fries	488	78.5	1.0	15.2	3.3	0.2
Mashed potatoes	185	34.8	2.9	2.1	1.6	1.3
Potatoes	168	36.8	2.1	0	0	0.4
Rice	265	54.8	0	2.5	0.6	0
*Desserts*						
Fruit* (orange, apple, pear, prune, banana)	35–86	9.2–19.6	6.5–16.5	0	0	0–0.001
Yoghurt*	55–100	7.8–17.0	7.4–16.8	0.1–1.3	0.1–0.8	0.2
Pudding*	104–154	16.3–28.9	13.1–27.6	2.4–3.8	0.5–2.5	0.2
Cookies*	108–248	13.9–32.1	7.3–18.9	5.3–12.3	1.9–6.4	0.1–0.4
Ice Cream*	33–150	8.1–22.0	7.9–15.2	0–9.5	0–4.7	0.02–0.2

*If different items were offered within the same food product type, ranges of nutritional values are provided.

### Design and intervention

**Fig 1:**
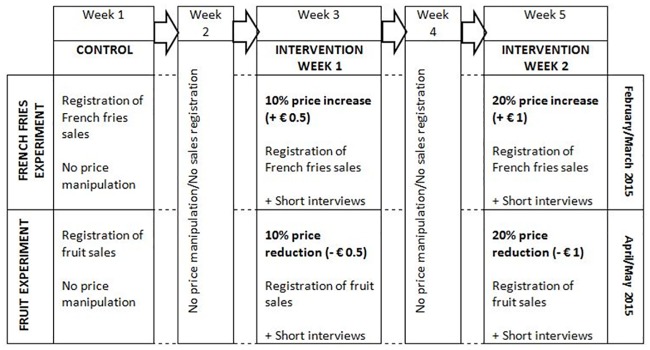
Flow-diagram of the French fries and fruit price experiments.

This mixed-methods study was a two-phased experiment designed to respectively examine the effect of 10 and 20% meal price increases (phase 1—French fries experiment) and 10 and 20% meal price reductions (phase 2—fruit experiment) on students’ French fries and fruit purchases. A flow-diagram of the experiment is provided in Fig 1.

#### Phase 1—French fries experiment

In February/March 2015, baseline sales data were collected during the first (control) week. During the third week (intervention week 1), students who chose menu 1, menu 2, health-menu or the vegetarian menu and chose French fries over rice or (mashed) potatoes, had to pay € 0.5 (= 10% of total meal price) extra. During the fifth week (intervention week 2), students had to pay € 1 (= 20% of total meal price) extra when choosing French fries. To avoid ‘meal’ bias (for certain meals French fries match better than do rice or (mashed) potatoes) the same menus were provided during control and both intervention weeks. In addition to the price manipulation, qualitative short interviews were performed in a convenient subsample of students during the two intervention weeks. In the second and fourth week, other menus were provided and no price adjustments were applied.

#### Phase 2—Fruit experiment

During the fruit experiment, which was conducted two months later than the French fries experiment (April/May 2015), the same five-week protocol was followed, except that students had to pay respectively € 0.5 (= 10% of total meal price) and € 1 (= 20% of total meal price) less during both intervention weeks when choosing fruit for dessert instead of yoghurt, pudding, cookies or other kinds of dessert. During control and both intervention weeks the same fruits (oranges, apples, pears, prunes, bananas) were offered. Although there was no standardisation of meals during the different conditions (because for the fruit experiment no meal bias was expected), we did standardise the different kinds of dessert being offered during control and both intervention weeks. Similar to the French fries experiment, qualitative short interviews were performed in a convenient subsample of students during the two intervention weeks.

#### Communication about the experiment

To make students aware of the price adjustments, several posters and information boards were placed at the entrance of the restaurant and at the cash registers. Also, two posters and one information board were placed at the stand where the French fries and/or fruits were served. Furthermore, a message about the experiments was posted on the online university intranet platform and on Facebook. During the French fries experiment, three Master students involved in this study helped serving the French fries wearing a special ‘French fries experiment’ t-shirt while providing additional information about the experiment when necessary. During both experiments, all communications (provided both in Dutch and in English) mentioned that the price had changed, and that this was a scientific experiment meant to help students make healthier food choices.

### Measures and procedure

#### Experiment

At the cash registers, students had to identify themselves with their student identification card so that no university employees, or other non-students would be included in the intervention. The chosen menus and whether or not French fries or fruit were chosen, along with the sex of the student, were registered. More specifically, cash register employees could choose between button 1, registering all students choosing a menu without French fries/fruit; button 2, registering all male students choosing French fries/fruit with their meal; or button 3, registering all female students choosing French fries/fruit with their meal. Due to practical reasons (cash register employees only have limited time for menu registration and payment), no other socio-demographics than sex could be registered at point of purchase. French fries and fruit sale counts relative to the total number of items sold were used as the outcome measures.

#### Short interviews

A semi-structured questionnaire was used to conduct short interviews in convenient subsamples (one for each intervention week) of students aged 17–25 years during lunch time at the on-campus restaurant. Each day of the intervention weeks (between 11:30 AM and 1:45 PM) one or two researchers were sent out to recruit as much students as possible. The answers to the questions were noted immediately and verbatim by the interviewer. The questionnaire included demographics, such as gender, age, residency, study discipline, but also height and weight in order to calculate BMI. The questionnaire also asked about their food choice and if and why the price manipulation had influenced their choice. Finally, it was asked whether they thought this kind of intervention (price adjustment) would change students’ French fries/fruit consumption in the long run and whether or not they believed this was a good initiative to help students make healthier food choices.

### Ethics statement

The study was approved (B.U.N. 143201421963) by the Medical Ethics Committee of the university hospital (Vrije Universiteit Brussel, Brussels, Belgium). Students verbally consented to participate in this study, as written consent was not required by the Medical Ethics Committee. Consents were not recorded or documented. The Medical Ethics Committee approved this consent procedure. All procedures followed were in accordance with the ethical standards of the responsible committee on human experimentation (institutional and national) and with the Helsinki Declaration of 1975, as revised in 2000.

### Data analyses

SPSS 23 was used to analyse French fries and fruit sales data as well as descriptive data obtained from the short interviews. Since French fries and fruit sale counts relative to the total number of items sold were used as the outcome measures, *Chi*^*2*^ tests (weight by count) were used to analyse differences in average French fries and fruit sales between control and both intervention weeks. Similar procedures were used to examine the moderating effect of gender. Short interview data were analysed using an inductive content analysis approach. In a first step, data (quotes) were examined for recurrent instances of some kind, which were then systematically identified across the data set, and grouped together by means of an open coding system [15]. In a second and third step, themes were derived from the data by grouping similar codes together into more general concepts (subcategories) and further categorising them into main categories. To ensure reliability of coding and data interpretation, analyses were carried out independently by two researchers. Doubts or disagreements were discussed with two other researchers until consensus was reached.

## Results

### Experiment

During the French fries price experiment, a total of 2,930 student consumers were registered during the control week, 2,344 during intervention week 1, and 2,325 during intervention week 2. During the fruit price experiment, a total of 3,235 student customers were registered during the control week, 3,802 during intervention week 1, and 3,728 during intervention week 2.

**Fig 2:**
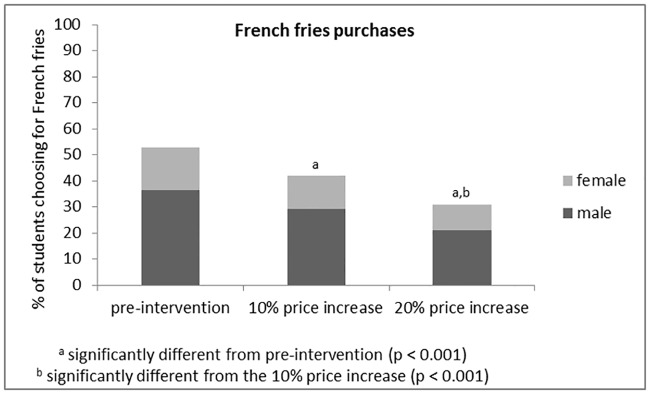
Changes in French fries purchases among university students.

**Fig 3:**
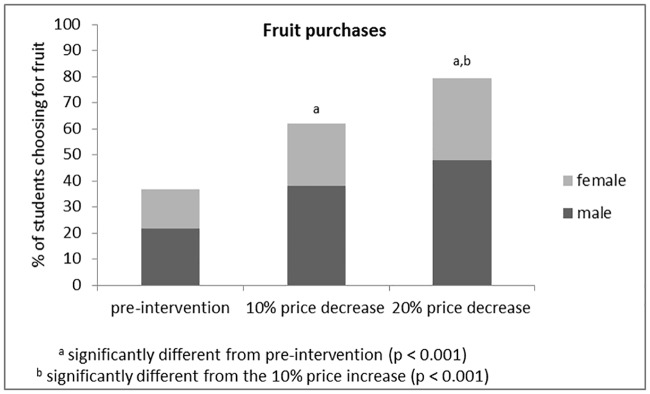
Changes in fruit purchases among university students.

Figs 2 and 3 show the respective differences in students’ French fries and fruit purchases between control and intervention weeks. In comparison to control week sales data (52.8% of all students eating lunch at the on-campus restaurant consumed French fries), significant decreases in French fries sales to respectively 41.9% (*chi*^*2*^ = 62.1, *p*<0.001) and 31.0% (*chi*^*2*^ = 250.1, *p*<0.001) were found during the first (10% meal price increase) and the second (20% meal price increase) intervention week. In financial terms, the respective price increases of 10 and 20% resulted in € 490.5 and € 721 profits. Also, French fries sales differed significantly between intervention week 1 and 2 (*chi*^*2*^ = 59.2, *p*<0.001). Gender proportion among students selecting French fries did not differ between control (68.8%) and both intervention weeks (69.6% males, *chi*^*2*^ = 0.2, *p* = 0.671; 67.8% males, *chi*^*2*^ = 0.2, *p* = 0.633).

Compared to the control week sales data (36.9% of all students eating lunch at the on-campus restaurant chose fruit for dessert), significant increases in fruit sales to respectively 62.0% (*chi*^*2*^ = 438.9, *p*<0.001) and 79.3% (*chi*^*2*^ = 1294.0, *p*<0.001) were found during the first (10% meal price reduction) and the second (20% meal price reduction) intervention week. Financially, the respective price reductions of 10 and 20% resulted in € 1,178.5 and € 2,958 losses. In addition, fruit sales differed significantly between intervention week 1 and 2 (*chi*^*2*^ = 273.0, *p*<0.001). Gender proportion among students choosing fruit did not differ between control (59.0%) and both intervention weeks (61.5% males, *chi*^*2*^ = 2.1, *p* = 0.146; 60.5% males, *chi*^*2*^ = 0.9, *p* = 0.355).

### Short interviews

Table 2 gives an overview of subsample characteristics and responses to the semi-structured questionnaire. During the French fries experiment, a total of 230 students were interviewed, consisting of 53.5% females, and having a mean age of 20.5 ± 1.9 years and a mean self-reported BMI of 21.7 ± 2.6 kg/m^2^. During intervention week 2, significantly more students indicated that the French fries price increase influenced their food choice (26.8 vs. 16.1%, *chi*^*2*^ = 3.9, *p* = 0.048), and more students (45.9 vs. 33.1%) believed that the price increase (when applied for a longer period of time) would have a long-term effect on their choice (*chi*^*2*^ = 4.0, *p* = 0.046) compared to intervention week 1. In contrast, less students (49.1 vs. 68.9%) thought that the price increase was a good initiative to help students make healthier food choices (*chi*^*2*^ = 6.3, *p* = 0.012), when comparing intervention week 2 with intervention week 1. Of those indicating that the price increase did not influence their food choice, 34.8% chose French fries, 8.3% chose rice, 9.4% chose potatoes, 7.7% chose mashed potatoes, and 39.8% chose other kinds of food (e.g. pasta or wok). Of those indicating that the price increase did influence their food choice, 14.3% chose French fries, 6.1% chose rice, 12.2% chose potatoes, 34.7% chose mashed potatoes, and 32.7% chose other kinds of food.

**Table 2:** Demographics and characteristics of the short interview subsamples (Mean ± SD, %).

	Subsample French fries experiment			Subsample Fruit experiment		
	Intervention week 1 (n = 118)	Intervention week 2 (n = 112)	Total (n = 230)	Intervention week 1 (n = 112)	Intervention week 2 (n = 115)	Total (n = 227)
Gender (% of females)	51.7	55.4	53.5	39.3	45.2	42.3
Age (years)	20.8 ± 1.9	20.2 ± 1.8	20.5 ± 1.9	21.0 ± 2.0	20.8 ± 2.0	20.9 ± 2.0
Study discipline (%)						
Human sciences	55.1	55.0	55.0	59.8	55.8	57.8
Exact sciences	22.0	26.1	24.0	25.0	30.1	27.6
Health and life sciences	22.9	18.9	21.0	15.2	14.2	14.7
Residency (% of students living in a student residence)	54.2	58.0	56.1	58.0	43.5	50.7
BMI (kg/m^2^)	21.7 ± 2.6	21.8 ± 2.6	21.7 ± 2.6	22.0 ± 3.0	21.8 ± 2.6	21.9 ± 2.8
Underweight (%)	10.5	4.6	7.7	6.4	8.0	7.2
Normal weight (%)	81.6	87.0	84.2	83.5	79.5	81.4
Overweight or obese (%)	7.9	8.3	8.1	10.1	12.5	11.3
Food choice (%)						
French fries	34.7	25.9	30.4	-	-	-
Rice	5.9	9.8	7.8	-	-	-
Potatoes	11.9	8.0	10.0	-	-	-
Mashed potatoes	12.7	14.3	13.5	-	-	-
Other	34.7	42.0	38.3	-	-	-
Food choice (%)						
Fruit	-	-	-	73.2	79.8	76.5
Yoghurt	-	-	-	2.7	2.6	2.7
Pudding	-	-	-	7.1	7.9	7.5
Cookie	-	-	-	6.3	3.5	4.9
Other	-	-	-	10.7	6.1	8.4
% of students indicating that the price adjustment influenced their food choice	16.1	26.8	21.3	38.4	42.6	40.5
% of students believing that the effects of a price adjustment would sustain in the long term	33.1	45.9	39.3	60.7	73.5	67.1
% of students believing that a price adjustment is a good initiative to help students make healthier food choices	68.9	49.1	56.1	94.6	93.9	94.3

During the fruit experiment, a total of 227 students were interviewed, consisting of 42.3% females with a mean age of 20.9 ± 2.0 years and a mean self-reported BMI of 21.9 ± 2.8 kg/m^2^. During intervention week 2, more students believed that the price reduction would have a long-term effect on their food choice compared to intervention week 1 (73.5 vs. 60.7, *chi*^*2*^ = 4.1, *p* = 0.042). No differences between both intervention weeks were found in the amount of students indicating that the fruit price reduction influenced their food choice (42.6 vs. 38.4, *chi*^*2*^ = 0.4, *p* = 0.518), nor in the amount of students believing that the fruit price reduction was a good initiative to help students make healthier food choices (93.9 vs. 94.6, *chi*^*2*^ = 0.1, *p* = 0.813). Of those indicating that the price reduction did not influence their food choice, 64.4% chose fruit, 3.7% chose yoghurt, 10.4% chose pudding, 8.1% chose some kind of cookie, and 13.3% chose something else (e.g. ice cream). Of those indicating that the price reduction did influence their food choice, 94.5% chose fruit, 1.1% chose yoghurt, 3.3% chose pudding, 1.1% chose another kind of dessert. None of these students chose cookies for dessert.

**Fig 4:**
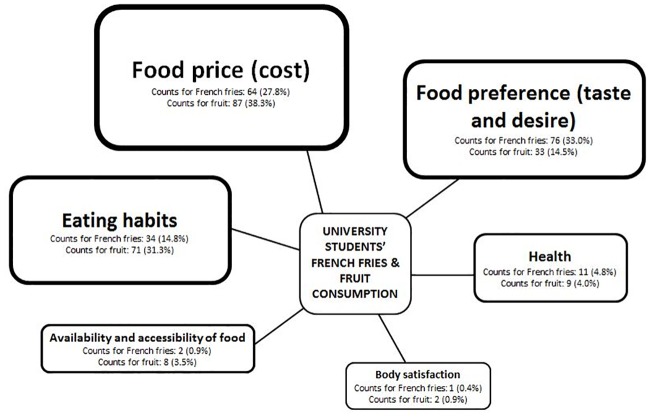
Factors influencing university students’ food choice during the experiment (accompanied by counts for both the French fries and fruit experiment, reflecting on how many students mentioned that factor during the short interviews).

A framework of factors influencing university students’ French fries and fruit purchases was developed (see Fig 4). Only commonly mentioned factors from the short interviews during both the French fries and the fruit price experiment were included in the framework. Next to price, the framework consists of five other factors, namely, food/taste preference, eating habits, health, availability and accessibility, and body satisfaction. Each factor is accompanied by a number of counts for the French fries experiment and a number of counts for the fruit experiment, reflecting how many students mentioned that factor during the short interviews. The most appropriate quotes were chosen to illustrate each factor.

#### Food price

Students were asked why the price manipulation had or had not influenced their food choice. Concerning the French fries price increase, some students declared they did not want to pay the extra amount for French fries, so they did not choose French fries: *“The price increase (of French fries) did not please me; I didn’t want to pay extra for French fries”*. Whereas the price reduction for fruit stimulated students to choose fruit for dessert: *“I chose fruit as dessert because I eat here (in the university restaurant) 3 to 4 times a week*, *and because of the price reduction I can save a lot of money”*. Students mentioned that such a price reduction may convince them to choose fruit, even when they planned to eat something else: *“I am short of money and I actually wanted to take a cookie*, *but the fruit price reduction convinced me (to take fruit)”*. In contrast, other students mentioned that price was not an issue for them: *“My parents pay for the food that I eat at university*, *which makes a price increase (during the French fries experiment) irrelevant to me”*. For some students it depends on the price increase rate: *“A price increase of € 0*.*5 is acceptable*, *but an increase of € 1 is too much”*.

#### Food preference

Next to price, food preference and taste were the most frequently mentioned factors among the students. Students reported that taste and desire towards a certain food product were important factors in influencing their food choices: *“It’s so tasty*, *I like French fries so much”*. Further, students mentioned that appeal or attractiveness of the food product is important to them: *“Fruit has to be appealing; if it (the fruit) doesn’t look good*, *then I’ll take something else”*. Students also expressed that their choice depends on which alternatives were offered: *“I don’t like the alternatives*, *I am not fond of mashed potatoes or rice*, *so I chose French fries”*. This was also illustrated by a number of students choosing the pasta or wok menu in which French fries, (mashed) potatoes or rice are not included: *“I just went straight to the lasagne; I even didn’t look at the other menus*, *because I really like lasagne”*. Students revealed that taste and desire are sometimes more important than price: *“If I really feel like having French fries*, *a price increase cannot stop me”*. Students also mentioned that it may depend on whether they feel like eating fruit at that particular moment: *“I eat fruit when I feel like eating fruit”*.

#### Eating habits

Students explained that their food choice was determined by their eating habits: *“I chose French fries because I always do”*. A similar statement was reported for fruit: *“I eat fruit every day”*.

#### Health

Many students mentioned that French fries were unhealthy which was a reason for them not to choose French fries: *“I think French fries are unhealthy*, *so I eat mashed potatoes because they are as tasty as French fries”*. Some students also revealed they consciously paid attention to how many times per day they should eat fruit: *“I eat fruit because it’s healthy and*, *according to the food pyramid*, *one should eat 3 to 4 pieces of fruit a day”*. Others try to avoid eating French fries during lunch when they know they will eat unhealthy foods in the evening: *“I didn’t choose French fries because I am going to eat unhealthy foods for dinner”*. In contrast, the aspect of health was sometimes used to approve eating French fries: *“I usually take rice*, *which is healthy*, *so now I may choose something unhealthy (French fries)”*. On the other hand, some students revealed they eat fruit to compensate for eating other more unhealthy foods: *“I chose fruit to be healthy and to compensate for eating French fries”*.

#### Availability and accessibility

Students who rarely eat French fries at home reported that it is an opportunity for them to eat French fries in the on-campus restaurant: *“I eat French fries because my parents do not often prepare French fries at home”*. Other students living in a student residence without access to a fryer indicated that availability of French fries in the on-campus restaurant stimulates them to choose this food product: *“I eat French fries because in my dorm room I don’t have a fryer”*. This home or dorm room availability could also have an effect on fruit consumption during lunch, namely, students may take another kind of dessert when eating in the on-campus restaurant because they regularly eat fruit at home or at the student residence: *“I already ate fruit around ten this morning and there is fruit available in my dorm room”*. Food choice may also depend on the available alternatives: *“I rather eat a cookie or ice cream (in comparison to fruit)”*. The other way around was also mentioned: *“I eat fruit when there are no better alternatives”*. Long waiting lines were also mentioned to be a barrier when choosing a certain food product: *“There was a long waiting line (for French fries)*, *so I did not take French fries”*. One student even indicated that the French fries price increase influenced the waiting line at the French fries stand and therefore influenced food choice in the other direction: *“Because of the price increase*, *less students chose French fries*, *so the waiting line for French fries was shorter*, *and because of that*, *I chose French fries*. *Otherwise*, *if I wanted to eat potatoes*, *I had to wait longer”*.

#### Body satisfaction

Some students explained they chose particular foods because they felt those foods helped them to achieve a desirable body shape: *“I try to watch my figure/weight*, *so I don’t eat French fries”*. Also, for some, body satisfaction is more important than price: “*The price manipulation did not influence my choice because I’m on a diet”*.

#### Other factors

Next to the abovementioned commonly mentioned factors during both experiments, some factors were only mentioned during the French fries experiment. A number of participants (n = 20) stated that menu/meal characteristics influenced their choice to eat French fries or not. Sometimes, French fries match better with a certain dish than the other starchy foods like rice or (mashed) potatoes: *“What else (other than French fries) should I eat with spare-ribs*? *I don’t think potatoes match this dish”*. Furthermore, one student mentioned hunger to influence French fries consumption: *“They (the university restaurant) were out of French fries (so I had to wait until a new load of French fries was prepared) and I was very hungry (so I chose to eat rice)”*. Factors only mentioned to influence the choice of fruits for dessert included weather (n = 1; e.g. choosing for fruit may depend on the weather conditions that day), and the origin of fruits (n = 1; e.g. whether or not fruits were organic).

Additionally, during both the French fries and fruit experiment, some students (n = 5 and n = 3 respectively) were influenced by the experiment itself. Students revealed that the duration of the experiment influenced their food choice: *“I know this (French fries) price increase only lasts for one week*, *so I don’t mind paying an extra amount of money for only a couple of days”*. In contrast, it was mentioned that the limited duration of the experiment influenced food choice in the opposite way: *“I won’t eat any French fries this week because of the price increase*, *but I will eat them when the price is down again”*. One of the students wanted to support the experiment and thought he/she was supporting it by eating French fries: *“I love to eat French fries*, *and I want to support this experiment”*. A similar reaction was found during the fruit experiment: *“I chose fruit for dessert because I support the initiative”*. Another student especially chose French fries to counter the experiment: *“Normally*, *I am on a diet*, *but as a counter-reaction against the experiment I chose to eat French fries”*. Lastly, one of the students expressed dissatisfaction about the experiment: *“I chose French fries because I am against discrimination; I don’t understand why this experiment is focused on students only and excludes staff members*. *One should better decrease the price of healthy foods”*. Finally, a number of students during the French fries experiment (n = 17) were not aware of the price manipulations: *“I had already chosen mashed potatoes before I realised the price of French fries had increased”*. Similar reactions were collected during the fruit experiment (n = 14): *“I didn’t know there was a fruit price reduction”*.

## Discussion

Both price increases and decreases (in combination with informing students about the aim of the experiment) were effective in changing students’ purchasing behaviour. A meal price increase of 10 and 20% when choosing French fries was associated with a respective 10.9 and 21.8% reduction in French fries sales. The fruit price reduction strategy resulted in an even greater effect; a meal price reduction of 10% when choosing fruit for dessert was associated with a fruit sale increase of 25.1%, whereas a price reduction of 20% was associated with a 42.4% increase in fruit sales. No moderating effect of gender was detected.

Compared to the study by Giesen and colleagues [14], in which a price increase of ≥25% on high-calorie foods successfully decreased the amount of calories purchased, we showed that smaller price increases (10 and 20%) can also influence students’ purchasing behaviour. Furthermore, our results are in line with studies in other populations showing that both taxation and subsidies for unhealthy and healthy foods respectively, can positively affect consumption of these foods. For example, a randomised controlled trial (RCT) by Waterlander et al. [16] among university students and staff showed that a VAT (Value Added Tax) increase from 6% to 19% (simulated in a three-dimensional web-based supermarket) led to 0.9 litre less sugar sweetened beverage purchases per household (or 400 ml corresponding 168 kcal per person) per week. The intervention had no significant effects on purchases in other beverage or snack food categories. A web based supermarket study by Nederkoorn et al. [17] showed that a 50% tax on high energy dense foods resulted in participants buying less calories (i.e. 16% decrease of high energy dense products), regardless of BMI and budget. With regard to price reductions, another RCT by Waterlander and colleagues [18] concluded that a 50% price discount on fruits and vegetables offered in Dutch supermarkets (real-life setting) was effective in increasing adults’ fruit and vegetable purchases by 3.9 kg per household per two weeks. In sum, pricing strategies may be recommended to health promoters, university policy makers and restaurant operators, when aiming to design and implement effective food-related health promotion interventions.

Results of the current study suggest that the price reduction of a healthy food product was more effective than the price increase of an unhealthy food product. Encouraging strategies were previously suggested to have greater effects on adolescents’ healthy eating behaviour compared to discouraging strategies [19]. Moreover, in comparison to other strategies like tax rises on unhealthy food items, discounting healthy foods or applying a lower VAT rate on healthy food was considered the most attractive strategy for Dutch adult consumers [20]. Subsidization of healthy foods, however, does not always seem to lead to decreased demands of unhealthy foods. An experiment, in which mothers responsible for their household purchases were given a certain budget to buy food items in an experimental room set-up as a grocery store, showed that making healthy foods cheaper (by 10%) may result in participants buying more unhealthy foods (+ 6.8%), and having higher energy intakes without changing the macronutrient profile of foods purchased [21]. Apparently, participants spent the money they saved on healthier foods on additional purchases of less healthy alternatives. Similarly, Waterlander and colleagues [22] observed that price discounts led to increased amounts of energy purchased, with the proportion of healthy foods being the same across 10, 25 and 50% discount levels. It was therefore suggested that a taxation strategy of unhealthy foods may have more favourable effects on participants’ dietary intake than subsidizing healthy foods [21]. The results of the current study should therefore be interpreted with caution. Since we did not measure energy or nutrient intakes of participants throughout the day/week, we cannot rule out any compensational behaviour of students. It may be, for example, that students choosing fruit for dessert used the amount they saved (€ 0.5 or € 1) to buy other less healthy (snacking or dinner) foods or beverages throughout the rest of the day/week. Future research in university students is needed to further investigate compensational food and beverage purchasing behaviour caused by food price manipulations.

In the present study, substitution effects were observed as a result of making meal prices more expensive (when choosing French fries) which makes it difficult to determine overall nutritional quality of purchases [8]. However, in the present experiment, the alternatives in the on-campus restaurant were limited to rice and (mashed) potatoes, pasta, wok or salad bar, with mashed potatoes being the most popular substitute (based on the observed food choice shift between students indicating the price did or did not affect their purchase behaviour). Nutrition analysis (data obtained from the university restaurant nutritionist and Nubel nutrition analysis software) revealed a much lower amount of calories, saturated fat and salt in one portion of mashed potatoes (185 kcal; 2.1 g fat of which 1.6 g saturated fat; 1.3 g salt per portion) compared to one portion of French fries prepared in vegetable oil (488 kcal; 15.2 g fat of which 3.3 g saturated fat; 0.2 g salt per portion). This suggests that overall nutritional quality of purchased meals may have improved during the experiment.

Along with price changes, it might be advisable to provide additional nutrition education in order to guide students in making well-advised and healthy food choices. Block and colleagues [23] showed that a 35% price increase in a hospital cafeteria caused a decrease in soft drink sales by 26%, while the sale decrease was even greater (36%) in combination with an educational campaign. Furthermore, a study by Michels and colleagues [24], investigating the effect of a 20% subsidy of healthy foods combined with education on food purchases in a college cafeteria, showed a greater increase (17% vs. 6%) in healthy food purchases after than during the intervention, suggesting that the nutrition education facilitated sustainability. Also the review by Niebylski and colleagues [10] highlighted that, alongside supportive pricing, education about healthy eating is a critical success factor to improve people’s food choices. The present qualitative results also showed that students care about their health and that health as such may be a driving factor towards healthy food choices. Thus, adding an educational component (e.g. providing health messages at point-of-purchase) to a pricing intervention in students may increase the overall effectiveness in the short and the long term.

In the present study, taste preference (next to price) was the most frequently mentioned factor explaining food choice. Taste and food preference have been shown to be important determinants of eating behaviour among university students [3]. Interventions combining pricing strategies with offering attractive and tasty food products may be more likely to succeed in improving students’ food choices. In accordance with other research [3, 11, 12, 25], students’ food choices were also influenced by the availability and accessibility of healthy foods. University restaurants are therefore challenged to offer healthy, tasty and meal matching starchy alternatives to French fries as well as a variety of fresh and appealing fruits.

A strength of the present study is its mixed-methods approach. This allowed us to understand why students did or did not change their food choices as a result of the price manipulations, but also which factors, next to price, explained food choice within the pricing experiment. Secondly, the current study used a real-life setting which maximises the external validity of the intervention. Thirdly, we anticipated that students would feel that certain meals matched better with French fries than with rice or (mashed) potatoes, as was mentioned during the short interviews. Therefore, during the French fries experiment, we excluded meal bias by providing the same menus during control and both intervention weeks. During the fruit experiment, we anticipated that availability of alternatives would influence students’ food choice (which was also mentioned during the short interviews), so we standardised the different kinds of dessert being offered during control and both intervention weeks. Fourthly, because it was shown that males may react differently to price adjustments than do females [13], we investigated the moderating effect of gender. Our results showed that price manipulations can be effective independent of gender.

A first limitation of the present study is that, because randomisation and a control restaurant were lacking, some internal validity concerns can be raised. Also, registering consumption in the other on-campus eateries would have allowed us to measure substitution effects on the restaurant level. Due to large differences in food availability between other university eateries, assigning a control restaurant was practically impossible. Assessing dietary intake, on the other hand, would have allowed us to appraise substitution effects on the intake level. It may be that changes in dietary intake during lunch affect dietary intake throughout the rest of the day. For example, Lachat and colleagues [26] demonstrated that giving university canteen customers (essentially students and university staff) two portions of fruits and one portion of vegetables for free at lunchtime, not only resulted in higher fruit and vegetable intake during lunch, but also in higher vegetable consumption during dinner and evening snacks. Another critique can be that it is unclear whether a ‘true’ effect was observed. For example, our qualitative data analysis showed that testing effects could not be ruled out (some students explained to be influenced by the experiment itself). Although, from a total of 457 short interview participants, only eight students revealed testing effects, it may be that, subconsciously, students’ food choices may have been influenced by the fact that they were part of an experiment. The validity of our experiment may thus be compromised by the communication strategy we used to announce the price modifications. However, qualitative results showed that knowledge about the experiment could influence students’ behaviour in any direction; e.g. one student did not mind to pay the extra amount of money on French fries for a couple of days, while the short duration of the experiment caused another student to wait for the experiment to be finished to eat French fries again. These complete opposite reactions to the experiment may even out possible bias. In addition, given this study’s relatively large effects, it seems unlikely that these effects are fully due to confounding factors. Nonetheless, we should interpret our findings with caution and take a possible overestimation of the observed pricing effects into account. A clear communication about the content and the duration of the pricing experiments was one of the conditions set by the Student Council prior to approval. Fourthly, it may appear that the price increase on the French fries led to a large decline in student customers (from 2,930 student customers in the control week to 2,344 and 2,325 in the two respective intervention weeks), while the price reduction on the fruit led to a large increase in student customers (from 3,235 student customers in the control week to 3,802 and 3,728 in the two respective intervention weeks). Although it is not clear whether these changes are related to the price changes they are important to consider as they have the potential to bias the sample. Since only a limited amount of information could be registered at the cash registers (asking too much questions at the cash registers would be too time consuming), no comparison could be made between characteristics of the control week sample and both intervention week samples. It seems likely, however, that a great part of the individuals were in the study during each week that it ran (including the quantitative and qualitative part of the study). Knowing to what extent we measured the same individuals during both experiments and during both quantitative and qualitative study arms, could have given us insight into which students were most susceptible (or not) to the intervention. In addition, if student consumers remained largely the same cohort across the two experiments, it may be that, after experiencing the first (French fries) experiment, students learned the underlying health message behind the experiment. If so, the results from the second (fruit) experiment may be influenced by the French fries experiment, which might partially explain the relatively large effect size found during the fruit experiment. Furthermore, short interviews revealed some students explaining that price did not matter to them (e.g. due to financial support of their parents). Hence, it could be that, compared to students with a higher socio-economic status, the effects of the price manipulations were greater among students with a lower socio-economic status. Unfortunately, due to the abovementioned practical limitations socio-economic status could not be assessed. Fifthly, we did not audiotape the short interviews because of practical reasons. We aimed to interview as much student customers as possible, while recording each interview could be deterrent for the respondents, possibly resulting in lower participation rates. Moreover, we anticipated that the noise made by the other student customers would interfere with the audio recordings. Sixthly, although qualitative data revealed that respectively 39.3 and 67.1% of students believed that the effect of a French fries price increase and fruit price reduction would sustain in the long term, the studied interventions were of short duration (one week each) and no follow-up measurements were conducted. Intervention periods of longer duration were not feasible, since the Student Council was already hesitant to approve two one-week interventions in which the price of French fries increased. It is therefore difficult to predict whether these intervention effects would sustain in the long run. In the abovementioned Dutch supermarket RCT by Waterlander et al. [18] the largest intervention effects (on fruit and vegetables) were observed at 6 months (end of the intervention), and disappeared 3 months after completion of the intervention. This suggests that, in case of continuous or permanent price reductions, the favourable effects of fruit price reductions may be sustained over a longer period of time. To confirm the latter in a student population, future research is needed to investigate the long-term effectiveness of such pricing strategies. Finally, in this experiment, combining both price reductions and price increases was not possible. It is possible that when combining both price manipulations, students would compensate the extra price they paid for French fries by choosing fruit for dessert (for which they had to pay less), resulting in a financial break-even effect.

## Conclusions

Both the meal price increase when choosing French fries and the meal price reduction when choosing fruit for dessert were effective in changing university students’ purchasing behaviour. More specifically, a 10 and 20% price increase was associated with a respective 10.9 and 21.8% reduction in French fries sales, while a 10 and 20% price reduction was associated with a respective 25.1 and 42.4% fruit sale increase. Pricing may be a promising strategy to improve university students’ eating behaviour. Next to price, there are other important determinants of students’ food choices, such as taste and desire, health, product access and availability. Hence, the likelihood of intervention success may increase when combining pricing strategies with offering healthy, tasty and meal matching starchy alternatives to French fries and offering a variety of fresh and appealing fruits.

## Supporting Information

S1 Fig Data French fries experiment. (XLSX)

S2 Fig Data fruit experiment. (XLSX)

S1 Table Semi-structured questionnaire French fries price experiment. (XLSX)

S2 Table Semi-structured questionnaire fruit experiment. (XLSX)
